# Learning Curve in Two-Port Laparoscopic Gastropexy Using FlexDex

**DOI:** 10.3390/ani14142016

**Published:** 2024-07-09

**Authors:** Federico Massari, Gary Matthew Martin Kelly

**Affiliations:** 1 DOCVET Clinica Veterinaria Nervianese, Via Rho 2, 20014 Nerviano, Italy; 2 Warren House Veterinary Centre, Lichfield Road, Walsall WS8 6LS, UK; garym.m.kelly@gmail.com

**Keywords:** gastropexy, veterinary laparoscopy, gastric dilatation and volvulus, canine laparoscopic gastropexy, minimally invasive surgery

## Abstract

**Simple Summary:**

Some large-breed dogs are at high risk of a life-threatening gastric dilatation and volvulus (GDV) when the stomach twists and cuts off the blood supply. This must be treated surgically with gastropexy. Gastropexy can be also performed before GDV occurs through open surgery or laparoscopically (keyhole). Laparoscopic gastropexy can be performed in various ways, and one of the newer trends is the use of semi-robotic instruments, such as the FlexDex device (Infiniti Medical, Brighton (US). This paper reports on a learning curve of a specialist surgeon with prior expertise starting to use the FlexDex device. Because this novel tool offers great range of movement, laparoscopic suturing is much easier with it, and within 12 cases, the operating time dropped by 73%, from 52 to 14 min. This is significant because many surgeons shy away from keyhole procedures due to the lengthy time to gain sufficient skills. Reducing surgical time also represents shorter, therefore, safer anaesthesia as well as lower costs of anaesthetic drugs and agents. Using advanced instruments decreases the technical difficulties related with keyhole surgery and laparoscopic suturing in particular.

**Abstract:**

Objectives: Keyhole gastropexy is becoming increasingly popular, and the new development facilitates shorter surgical times. This paper reports on the learning curve in two-port laparoscopic gastropexy using FlexDex in a specialist’s hands. FlexDex is a novel tool combining aspects of robotic surgery without requiring an expensive robot theatre setting. Methods: Cohort of 16 dogs >25 kg and at high risk of gastric volvulus and dilatation (GDV) undergoing elective laparoscopic gastropexy were enrolled in the study consecutively from 5/2022 to 9/2023. All patients were operated on by one surgeon (FM), and surgical time was recorded to assess learning curve. Competence was defined as plateauing surgical time. Detailed follow-up at 1 day, 7 days, 14 days, 2 months, 6 months, and long-term was recorded for success rate and complications. Ultrasound examination was scheduled at 4–6-month review to confirm lasting success of the gastropexy. Results: All 16 patients were operated on successfully without any significant complications, as confirmed on the ultrasound. The surgical time of laparoscopic gastropexy reduced from 52 to 14 min (reduction of 38 min/73%) and reached plateau after the 12th case of the 16, making it a very steep learning curve in specialist hands. There were no serious complications, and success rate was 100% at the 6-month ultrasound assessment. Clinical Significance: This is the first paper to report on the learning curve with the FlexDex device in a two-port laparoscopic gastropexy setting. It effectively halves the operating time to 30 min, making the surgery safer for the patient and more cost-efficient, without compromising the result.

## 1. Introduction

Gastropexy was performed in veterinary medicine for the first time in 1971 [1] and it was traditionally performed via open access. In 1979, it was introduced as surgical treatment for gastric dilatation and volvulus (GDV). The recurrence rate of GDV without gastropexy was reported as high as 80% compared to 5% risk with gastropexy [2] and the lifelong risk of GDV in high-risk breeds was up to 36.7% [3]. The median age at which GDV first occurred was 5 years and the median age of death from GDV was 7.92 years [4], suggesting artificially shortened life expectancy in high-risk breeds, although it has been reported that 80% of patients with GDV survive the emergency surgery [5]. It is only natural that veterinary surgeons started to consider prophylactic gastropexy rather than wait for a GDV to occur. Scientific reports on the technique and complications in prophylactic gastropexy started to appear in the 1990s [6,7,8], and with the rise in laparoscopic surgery, the latest research papers focus mostly on the minimally invasive techniques [9,10]. Apart from surgical complications, there does not seem to be any adverse effect on related bodily functions such as gastrointestinal transit time [11].

There is no doubt about the benefits of laparoscopic approach for the patient, such as shorter recovery and better pain score [12], but the introduction of keyhole procedures has always been hindered by the set-up cost as well as longer learning curves. The lack of standardised training and limited use of simulation teaching for veterinary surgeons meant that the progress of minimally invasive surgery has had a slower uptake compared to human healthcare [13,14]. With the lack of training and support, many surgeons also chose to perform laparoscopically assisted operations [15]. Based on the published data, laparoscopically assisted gastropexy may seem to have similar outcomes to total laparoscopic gastropexy [16], but it is hard to assess significant complications with a reported incidence in humans 14:10,000 [17] in a canine cohort of less than 30.

To fight off sceptics, there has been an even bigger push to adopt inventions that make laparoscopic procedures quicker and safer, notably the introduction of robotic surgery. The Da Vinci robot was approved for clinical use by the FDA in 2000 [18], but due to its excruciating price (GBP 2 million), its use in veterinary surgery is anecdotal [19]. There are however routine endoscopy systems that allow 3D view with special glasses [20], and there has been a development in specialised tools to allow wrist joint-like manoeuvres. One of the newer tools is FlexDex, which is a hand-held needle holder. It uses a system of pulleys and cables, allowing wide range of movements and since it is non-electrical its cost is around GBP 500, making it more accessible.

The aim of this study was to assess the introduction of FlexDex in clinical practice, focusing on the learning curve in specialist hands, defined as plateauing reduction in theatre time [21]. The alternative measurement of learning curve by reduced complication rate did not seem appropriate given little to no complications reported in the literature on such small cohorts [22]. To the authors’ knowledge, this is the first study to date to describe implementation of this novel tool and related learning curve, although the use of this device has been previously described in veterinary medicine with [23].

## 2. Materials and Methods

### 2.1. Study Design and Inclusion Criteria

This study was a prospective observational real-life field study from a referral centre with specialist level of expertise for carrying out similar procedures. The participants were recruited in the 16-month period from 5/2022 to 9/2023 to allow sufficient time for follow-up. All dogs weighed over 25 kg and were from selected breeds at high risk of gastric dilatation and volvulus (GDV) (Table 1), and gastropexy procedure was offered on its own or as a part of other procedures, mostly neutering, and those whose owners agreed were enrolled in the study consecutively. Apart from the above inclusion criteria, authors did not set further inclusion criteria, but by design, the cohort was healthy and young with ASA status 1 [23], and the procedure was elective.

### 2.2. Protocols

**Figure 1:**
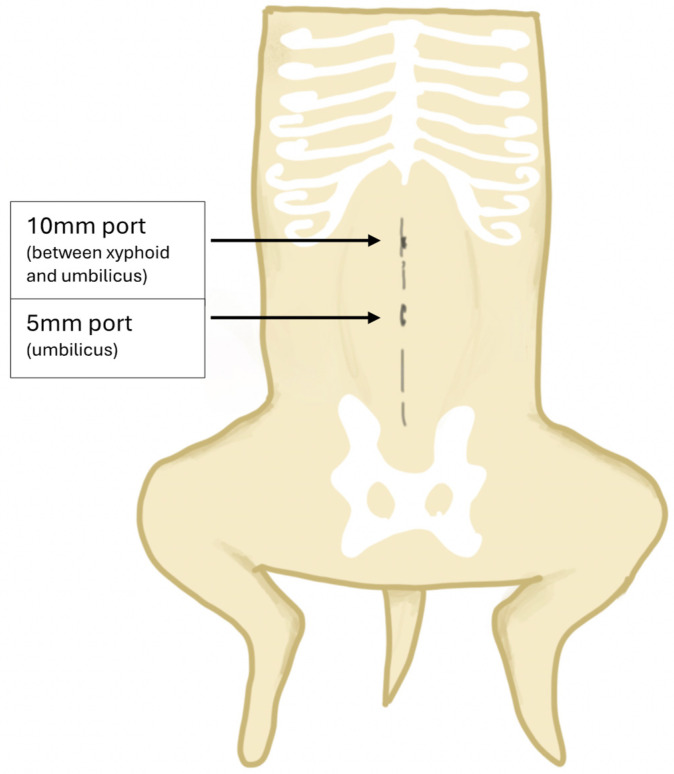
Placement of ports demonstrating two-port technique: Camera port for 5 mm trocar at the umbilicus and main operating port 10 mm between the umbilicus and xyphoid.

All patients underwent laparoscopic gastropexy on its own or as a part of neutering. All procedures were performed by the same surgeon (FM) to provide a clear learning curve in specialist hands. A two-port technique was used (Figure 1) since it has the shortest surgical time and significantly lower pain score compared to single- and three-port techniques [25]. FM had prior expertise in two-port laparoscopic gastropexy, and the only new element was the use of FlexDex, which was anticipated to speed up the surgery. The surgeon used barbed sutures and continuous suturing to secure the stomach to the abdominal wall after superficial scarification, which is a technique described previously in the literature [1,26,27].

All gastropexies were performed in the same manner: First port (5 mm) was inserted under video guidance, using Ternamian-threaded cannula placed in the umbilical region (Figure 1). Abdomen was inflated to 10–12 mmHg to allow comfortable visualisation and safe placement of the second port. Second port placement was under direct visualisation between the umbilicus and xyphoid (10 mm port). Using 30° camera, surgeon checked the gastropexy site, close to the insertion of the diaphragm, approximately 3–5 cm lateralised from the linea alba. The choice of target area on the stomach was selected by pulling the stomach posteriorly and evaluating, by reducing the intra-abdominal pressure to 4 mm/Hg in order to have no tension on the hepatogastric ligament. Stomach wall was suspended with a suture through the abdominal wall and target surface area of gastric serosa, and peritoneum was scarified with diathermy. Suturing was performed with a barbed wire (V Lock or Stratafix) using FlexDex device (Infiniti Medical, Birghton (US) allowing 360° movements of the instrument. After placing the initial loop, surgeon performed 5–8 throws in continuous stitching, obtaining at least 4–5 cm of joint-sutured tissue.

The main outcome of the study was the surgical time required to complete the gastropexy, counted from the insertion of the barbed wire through the port to the completion of gastropexy. In cases undergoing joint procedures (mostly ovariectomy as well as gastropexy), gastropexy was performed second after the technically easier procedure. Learning curve was measured as the progressive shortening of the surgical time, and consistent plateau of surgical time was regarded as the end-point of the learning curve, defining number of procedures necessary to achieve competence in two-port laparoscopic gastropexy using FlexDex in specialist hands.

Authors also recorded complications and success rates of the operation. Complications were recorded in the following intervals: intraoperative, immediate postoperative, and in the first 7 days, 14 days, 2 months, 6 months, and long-term follow-up. Ultrasound was performed between 4 and 6 months postoperatively to confirm ongoing attachment of the stomach to the abdominal wall, similar to the design of previous publications [28].

### 2.3. Data Analysis

Surgical times were reported in a simple observational manner as minutes required for completion of the procedure and percentage in time reduction. Demographics were reported in generic numbers, percentages, averages, and medians, and complications were recorded in generic numbers and percentages from the total.

## 3. Results

### 3.1. Patients

During the time period, 16 patients were included in the study consecutively with no exclusions. Cohort consisted of 12 female and 4 male dogs with average age 2.5 years (median 2.4 years). The average weight was 42.1 kg (median 39.5 kg, SD 11.0). The represented breeds were Leonberger (4/25%), Irish Setter (2/12.5%), Ridgeback (2/12.5%), Labrador Retriever (1/6.25%), English Setter (1/6.25%), German Shepherd (1/6.25%), Maremmano (1/6.25%), Corso (1/6.25%), Rottweiler (1/6.25%), Terranova (1/6.25%), and Weimaraner (1/6.25%).

### 3.2. Treatments

There were no cases (0%) requiring conversion to open surgery. Barbed suture was used in all cases although the first four cases were performed using V-Loc 0, which went out of stock temporarily; hence, FM continued using Stratafix 0 or 2-0 (Ethicon, Johnson & Johnson, Somerville (Raritan, NJ, USA)). Five participants (31.25%) had only gastropexy while ten participants (62.5%) underwent laparoscopic ovariectomy in the same setting. One patient had laparoscopic ovariectomy, gastropexy, and a removal of an intercostal foreign body.

### 3.3. Learning Curve (Outcomes)

**Figure 2:**
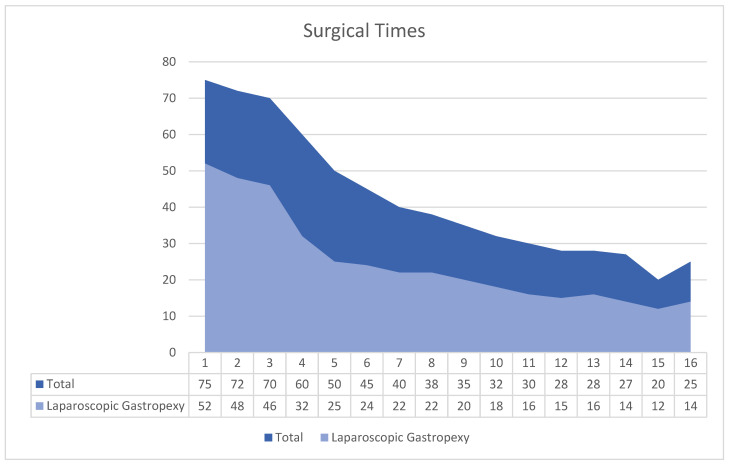
Outcomes of the learning curve—total surgical time and time required for laparoscopic gastropexy (shown in minutes).

Laparoscopic gastropexy on its own took initially 52 min, reducing to as little as 12 min, plateauing at 14–15 min after the 12th case. This represents a reduction in operating time of 38 min/73%. Total surgical time reduced as well although when analysing cases with a second procedure, the results were not consistent (Figure 2).

**Figure 3:**
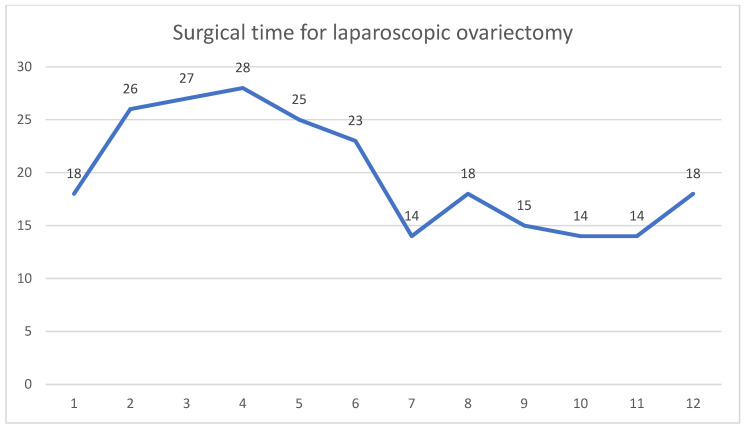
Surgical times for laparoscopic ovariectomy, calculated as total time of surgery with subtracted time for gastropexy in minutes. These results are further commented on in the discussion section.

Among the ovariectomy cases, there was a reducing trend in the surgical time of the ovariectomy, ranging between 14 and 28 min (median 18), but it was not consistent, and overall, there did not seem to be such a great benefit of using FlexDex in reducing the operating time (Figure 3).

FM encountered some technical challenges (Table 2) mostly originating from the placement of the cranial 10 mm port. When placed on the linea alba, it interfered with the device manipulation at the end of the suture line due to its close proximity to the gastropexy site and a lack of manoeuvring space. When FM moved the port placement to the patient’s right, it increased the manipulation space but, in some cases, the falciform fat obstructed the view.

There were no serious complications reported in any patients short-term or long-term (Table 3).

## 4. Discussion

This observational study is the first to date to report on the learning curve in two-port laparoscopic gastropexy using FlexDex and offered valuable insight on advantages and disadvantages of this device in laparoscopic suturing. There was a 73% reduction in surgical time (38 min) within the first 12 cases of the 16-dog cohort (Figure 2). Reduction in surgical time of laparoscopic ovariectomy (Figure 3) in 12 non-consecutive cases alongside the gastropexy was not as consistent and did not show the typical shape of a learning curve.

This paper is a first reporting to date on the use of the FlexDex device in laparoscopic gastropexy in dogs. The study benefited from a robust methodology with a clear follow-up pathway to detect complications in regular intervals. Another strength was the prospective design and academic support of a referral hospital. This article adds data into the growing body of evidence on laparoscopic procedures in dogs.

Regarding limitations, it can be argued that by including only 16 patients and seeing plateauing surgical times around 12 cases (15 min) with a little one-off reduction at the 15th case (12 min), the authors have not fully explored the learning curve, and given further 20 cases, the average surgical time would be even shorter by a few minutes. The reported surgical time for two-port laparoscopic gastropexy in the literature was 18 min (13.6–22.4 min) with the use of conventional laparoscopic equipment [25]. It is arguable that this was not a learning curve study, and these were the ‘final’ times by a competent surgeon after sufficient experience and training. Nonetheless, the reported average time was 4 min longer than the plateau time in this study, representing a 22.2% reduction in operating time. Another limitation is the isolated focus on the reduction in surgical time of gastropexy alone, while in clinical practice, it will be frequently performed as joint procedure with laparoscopic spay, where the benefit of FlexDex does not seem that significant. Put in a wider perspective, while the reduction in surgical time for laparoscopic gastropexy was significant, when added to another procedure time, it will represent lesser percentage from the total surgical time of the joint procedure. Lastly, it can be argued that the curve was described on one selected approach (scarifying, two-port technique), potentially limiting generalisation to modified techniques, such as one- and three-port approaches and non-scarifying gastropexy [29].

The above limitations together with the advantages will ultimately determine if veterinary surgeons choose to adopt this novel tool. This study demonstrates that there is a benefit of using FlexDex for procedures requiring laparoscopic suturing and as such leads to shorter surgical times, making the anaesthetic time shorter hence less risky for the patient. Laparoscopic suturing is avoided by many surgeons due to its complexity and long learning curve and this could be one of the ways to avoid the initial struggles. It could also encourage the induction of more complex laparoscopic procedures in veterinary specialist centres.

Future research should expand on the above findings and determine the learning curve among more surgeons, ideally including specialists as well as laparoscopic beginners. It may be possible that the benefits to junior vets will be even more prominent.

## 5. Conclusions

These results demonstrate that FlexDex has a clear benefit in technically challenging laparoscopic operations, such as those requiring intracorporeal suturing. The learning curve is steep, and in specialist hands, within the first 12 cases, the surgical time is reduced by 73%, representing a reduction in anaesthetic risks to the patient as well as more cost-efficient surgery. It may be arguable whether this benefit is significant in less technical procedures (i.e., without laparoscopic suturing), such as a simple ovariectomy where use of ‘linear’ tools results in similar operating times.

Authors accept that the presented findings are limited to one surgeon only, with prior expertise, so the learning curve only applies to the introduction of FlexDex. The study was planned for a specific time period, but it is possible that given further number of cases the time might drop even further, although possibly not that significantly.

## Figures and Tables

**Table 1:** Selected breeds with high risk of GDV who were offered participation in the study based on prevalence [4] and odds ratio compared to low-risk breeds [24].

Breeds	Prevalence of GDV	Prevalence of Death due to GDV	Odds Ratio
Great Dane			42 OR
Grand Bleu de Gascogne	21.4%	50%	
Bloodhound	14.3%	30.5%	
Otterhound	9%	7.4%	
Irish Setter	7.2%	5.3%	14.2 OR
Bracco Italiano	5.3%	Not available	
Weimaraner	5%	11.6%	19.3 OR
Saint Bernard	4.6%	15.1%	21.8 OR
Italian Spinone	3.6%	6.4%	
Akita	3.5%	10.7%	

**Table 2:** Intraoperative technical issues encountered during laparoscopic gastropexy.

Issue		Issues
Visibility	Case 1	Falciform fat on FlexDex
	Case 11	Falciform fat of FlexDex
Manoeuvring	Case 3	Tip of FlexDex too close at the end of the suture
	Case 10	Tip of FlexDex too close at the end of the suture
	Case 16	Big (Large) falciform fat conflicting with the FlexDex at the end of the suture

**Table 3:** Complications by timeframe.

Time	Complications
Intraoperative	None
Early recovery (up to 7 days)	Minor skin irritation (3/18.75%) Wound disruption after patient licked the wound
Middle-term (7–14 days)	Nodule at port site (1/6.25%)
2-month follow-up	None
6-month follow-up (ultrasound)	None (USS confirmation of successful surgery 16/100%)
Long-term follow-up	None Lost in follow-up (1/6.25%)

## Data Availability

The raw data supporting the conclusions of this article will be made available by the authors on request.
